# Supplementary material for: General prediction of T cell receptor antigen specificity from sequence using AlphaFold 3
Source: bioRxiv. 2026 Jun 4:2026.06.02.729478. Preprint. [Version 1] doi: 10.64898/2026.06.02.729478 (PMC13252052; doi:10.64898/2026.06.02.729478)
Supplement: Supplement 1 [file NIHPP2026.06.02.729478v1-supplement-1.pdf]

bioRxiv preprint doi: https://doi.org/10.64898/2026.06.02.729478; this version posted June 4, 2026. The copyright holder for this preprint (which was not certified by peer review) is the author/funder, who has granted bioRxiv a license to display the preprint in perpetuity. It is made available under a CC-BY 4.0 International license.

Supplementary Figures

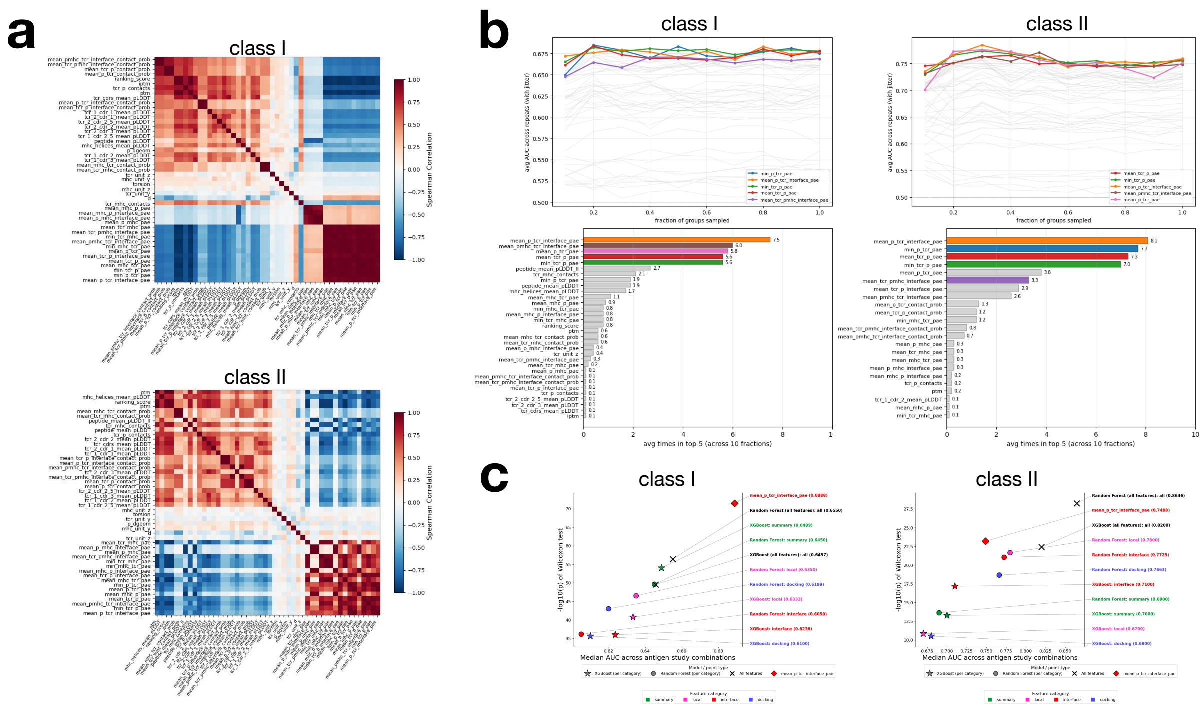

Figure S1: **Stability of individual feature performance within the IEDB training data, and comparison against alternative machine learning models. (a)**Feature-feature correlation analysis for the IEDB dataset shown in Figure 3, calculated based on Spearman comparisons. **(b)** Stability of the features described in Figure 3 across randomized subsamples of the IEDB dataset at increasing fractions of the available data (upper plots, x-axis). At each fraction, we generated 10 independent random subsets, ranked all features according to their median antigen-study-combination AUC, and then averaged the results across runs (lower plots). **(c)** Performance of Random Forest and XGBoost classifiers trained on the IEDB dataset. Models used either all features within a particular class (denoted using the color scheme of Figure 3), or all features within all classes (black). Models were trained and evaluated using 5-fold cross-validation with folds defined such that all cognate and non-cognate triads associated with a given antigen-study combination were assigned to the same fold. Performance was evaluated using the same antigen-study-combination ROC framework used in Figure 3. In all panels, the feature selected for downstream analysis (AF3-PTI-PAE) is named "*mean_p_tcr_interface_pae*"; it was added to panel (c) as a point of comparison (red diamond).

bioRxiv preprint doi: https://doi.org/10.64898/2026.06.02.729478; this version posted June 4, 2026. The copyright holder for this preprint (which was not certified by peer review) is the author/funder, who has granted bioRxiv a license to display the preprint in perpetuity. It is made available under a CC-BY 4.0 International license.

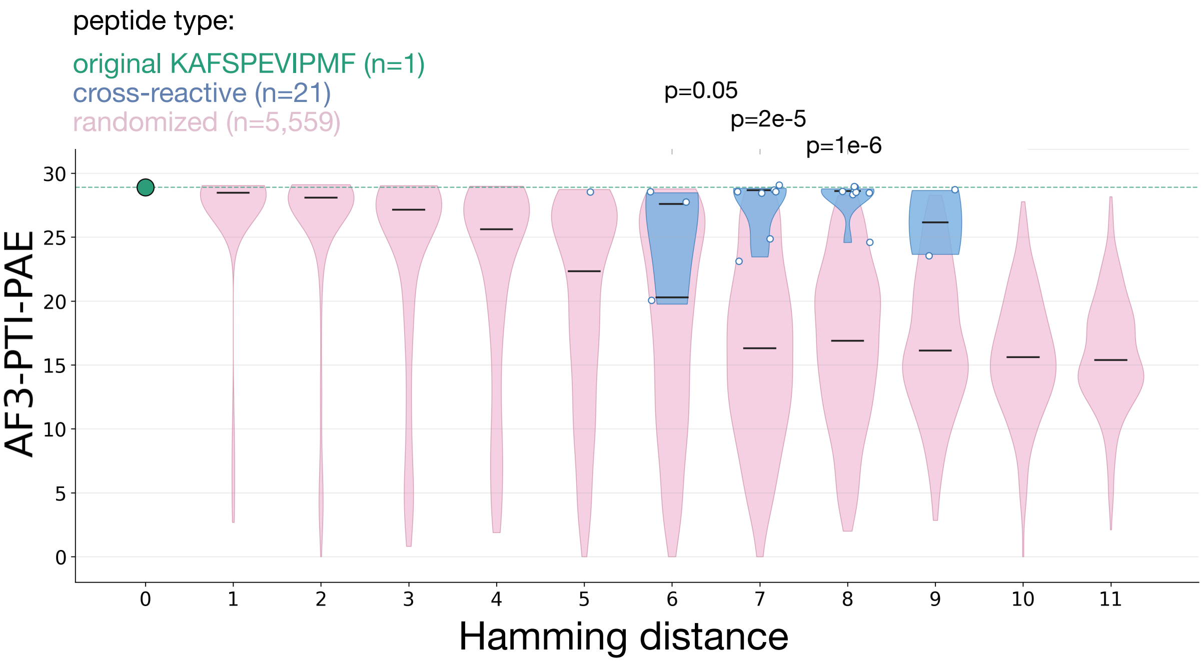

Figure S2: **AF3-PTI-PAE analysis of peptide cross-reactivity to a fixed TCR**. A set a 21 peptides, identified previously using yeast display [43] as being recognized in the context of HLA-B*27:03 by the HIV-specific TCR "AGA1", were the largest set of cross-reactive antigens for a single TCR in our IEDB dataset. Shown for the original HIV peptide (green), the 21 cross-reactive peptides (blue), and 4,559 controls randomly-mutated to a range of distances (pink), are the AF3-PTI-PAE scores for the corresponding triads (*y-axis*), plotted as a function of Hamming distance from the original peptide (*x-axis*). For the 3 bins containing ≥3 cross-reactive peptides, p-values were calculated using Wilcoxon Rank-Sum tests.

bioRxiv preprint doi: https://doi.org/10.64898/2026.06.02.729478; this version posted June 4, 2026. The copyright holder for this preprint (which was not certified by peer review) is the author/funder, who has granted bioRxiv a license to display the preprint in perpetuity. It is made available under a CC-BY 4.0 International license.

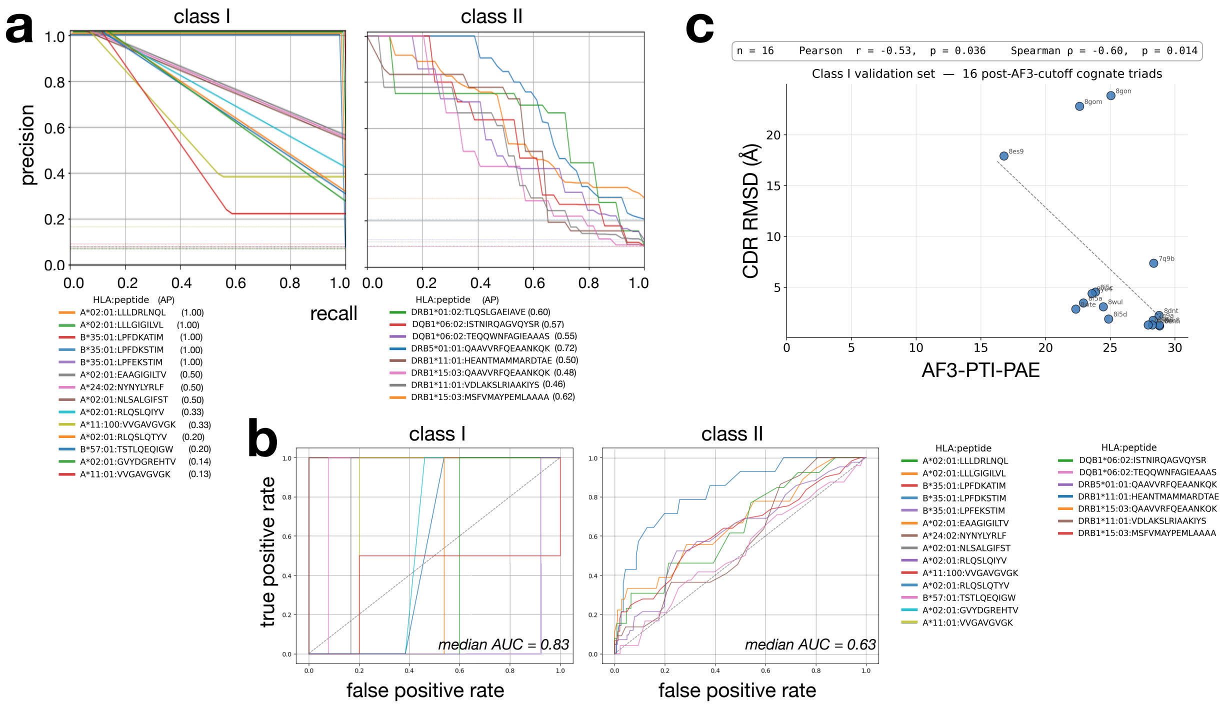

Figure S3: **Further analysis of the validation dataset from Figure 5.** **(a)** Antigen-centric Precision-Recall analysis of the class I and II validation triads. AP denotes Average Precision. **(b)** Antigen-centric ROC curves showing the performance of the *Random Forest (all features)* model described in Supplementary Figure 1c, on the validation dataset. **(c)** Correlation between AF3-PTI-PAE model score and structural accuracy (CDR RMSD) for all validation triads with available crystal structures. PDB identifiers are shown next to each datapoint.
